# Case Report: Sustained weight loss and glycemic control from repeated long-term fasting in type 2 diabetes

**DOI:** 10.3389/fcdhc.2025.1572245

**Published:** 2025-08-07

**Authors:** Marie Knufinke, Marco Lebbing, Robin Mesnage

**Affiliations:** ^1^ Science Department, Buchinger Wilhelmi Clinic, Überlingen, Germany; ^2^ Department of Health Sciences and Technology, ETH Zurich, Zurich, Switzerland; ^3^ Department of Nutritional Sciences, School of Life Course Sciences, Faculty of Life Sciences and Medicine, King’s College London, London, United Kingdom

**Keywords:** type 2 diabetes mellitus, fasting, weight reduction, glycemic control, case report, food reintroduction, remission, microbiome

## Abstract

Type 2 diabetes mellitus (T2DM) is a common metabolic disorder typically managed with medication; however, fasting has recently attracted attention for its potential benefits in glycemic control, weight management, and even potential remission. This case report examines the effects of repeated long-term fasting on weight reduction, glycemic control, and medication requirements in a 57-year-old man with T2DM. The patient, who had a history of inadequate glycemic control despite conventional treatment, opted for repeated long-term fasting under medical supervision. He completed several fasts ranging from 11 to 20 days each, with each fasting period followed by a gradual reintroduction of food via a hypocaloric lactovegetarian diet (800–1,800 kcal) over 4 to 16 days. The intervention resulted in sustained weight loss and improved blood sugar control. Notably, clinically meaningful improvements occurred in fasting blood glucose levels, which necessitated adjustments in his antidiabetic medications. Enhanced insulin sensitivity was evidenced by decreased HbA1c levels and a reduced dependence on hypoglycemic agents. Additionally, post-fasting evaluations indicated improvements in inflammatory markers and a reduction in fatty liver disease. In summary, repeated long-term fasting in this patient was associated with sustained weight loss, improved glycemic control, and reduced medication requirements, thereby enhancing the overall management of T2DM. Further research, including randomized controlled trials, is needed to better understand the long-term safety and effectiveness of this intervention.

## 1 Introduction

Type 2 diabetes mellitus (T2DM) is a chronic metabolic disorder which is predicted to affect over 783 million by 2045 (1). Management of T2DM typically involves lifestyle modifications, pharmacotherapy, and insulin supplementation as the disease progresses (1). However, most of these therapeutic strategies for treating T2DM effectively maintain glucose levels and avoid – or at least limit – the related complications, but they do not target the underlying causes of the disease (2). Consequently, achieving and maintaining optimal glycemic control remains a significant challenge for many individuals with T2DM.

Obesity and type 2 diabetes are major modifiable risk factors for cardiovascular disease, amplifying the risk through chronic inflammation, endothelial dysfunction and altered growth hormone secretory capacity (3, 4). Although controlling multiple risk factors (lipids, blood pressure, glycaemia, body weight, smoking) can reduce cardiovascular events by at least 50%, fewer than 20% of patients meet all treatment targets (5). This underscores the importance of developing novel interventions to curb cardiovascular disease in T2DM.

Fasting, the voluntary abstinence from food and caloric beverages, has been practiced for centuries for various cultural, religious, and health-related reasons (6). In recent years, fasting has garnered interest for its potential therapeutic effects on metabolic health, including weight loss, improved insulin sensitivity, and glycemic control (7).

An increasing number of scientific studies suggest that fasting could help treat T2DM and leads to remission (8–10). This is supported by the rapid reversal of hepatic insulin resistance in response to a sudden decrease in calorie intake (11, 12), and by cases showing that long-term remission of type 2 diabetes can be achieved by weight loss and reduction in excess visceral fat (2, 8).

This case report presents the effects of repeated long-term fasting in a 57-year-old man with T2DM. The case adheres to the CARE (CAse REporting) guidelines for transparent and comprehensive reporting of individual cases in medicine.

## 2 Case description

In 2019, a 57‐year‐old US American male of Bangladeshi descent reported a 10‐kg weight gain over the past decade with an initial weight of 93.7 kg (BMI 30.6 kg/m²). He reported persistent lethargy and lack of regular exercise, with poor glycemic control (HbA1c 9.7%) despite ongoing metformin and glipizide therapy. His comorbidities included dyslipidemia (managed with rosuvastatin 10 mg) and hypertension (diagnosed in 2009, treated with valsartan 40 mg daily).

Type 2 diabetes mellitus (T2DM) was first diagnosed in 2011 when his weight was approximately 86–90 kg, and he was started on low‐dose metformin along with weight loss counseling. Despite these measures, his glycemic control worsened, prompting an increase in metformin to 850 mg twice daily in 2014 and the addition of canagliflozin, later switched to glipizide 5 mg twice daily due to hyperkalemia. Although he intermittently pursued 5‐day fasting mimicking diets and protein shake regimens with initial weight loss, the weight was eventually regained.

A strong family history of metabolic disorders was noted on his maternal side across at least two generations, with his mother, maternal grandmother, and extended maternal relatives affected by T2DM, hypertension, and hyperlipidemia. His mother died at 72 from idiopathic thrombocytopenia, his father at 68 from gallbladder carcinoma, and his younger sister has been diagnosed with diabetes, hyperlipidemia, hypothyroidism, and food intolerances.

## 3 Diagnostics and outcomes

The patient followed a protocol of repeated long-term fasting followed by periods of reduced caloric intake as described in peer-reviewed guidelines (13). Among different fasting protocols (14), Buchinger fasting was used in order to induce a substantial calorie deficit known to normalise glycemic control in other studies (15). In brief, during fasting, his caloric intake was limited to a maximum of 250 kcal per day, supplied as vegetable soup, fruit/vegetable juices, and yogurt—along with electrolyte supplements (3 capsules three times daily, each capsule containing 345.48 mg of calcium citrate, 174.15 mg of potassium citrate, 361.11 mg of magnesium citrate, 18.12 mg of medium-chain triglyceride oil, and 4.27 mg of zinc citrate). The food reintroduction phases were calorie restricted (from 800 kcal to 1600 kcal per day), consisting of an ovo-lacto-vegetarian organic diet with a progressive increase of energy from 800 to 1600 kcal/day. Once each food reintroduction was completed, he resumed a habitual omnivorous diet during the intervals between fasting cycles. Additionally, cholecalciferol (vitamin D_3_, 20,000 IU) was administered during the fasting periods as part of the supportive medical regimen and was continued thereafter as a maintenance supplement.

**Figure 1:**
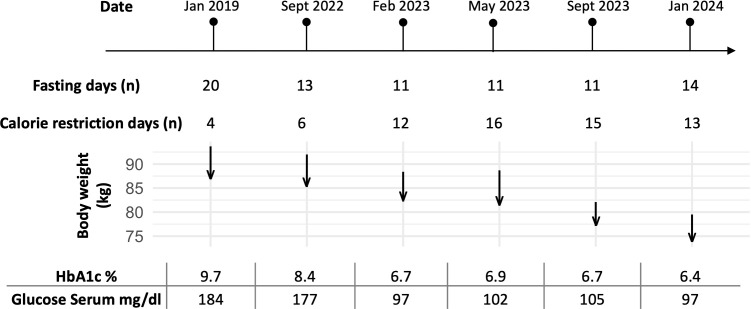
Timeline of the patient’s fasting periods and corresponding changes in body weight (kg), HbA1c (%), and glucose serum levels (mg/dl) over six documented stays from January 2019 to January 2024. The number of fasting days for each stay is indicated, along with arrows showing the patient’s body weight changes during the fasting period (beginning of the arrow, start weight; tip of the arrow, end weight). The duration of each fasting stay was tailored to the patient’s availability, while the food reintroduction period was adjusted based on well-being and motivation to ensure tolerability. HbA1c and glucose serum levels at baseline before fasting demonstrated metabolic improvements with repeated fasting periods.

The fasting duration ranged from 20 days in 2019 to 11–14 days in 2022 to 2024 (**Figure 1**). During this time, he experienced a progressive reduction in body weight, with a total weight loss of 22% of his initial body weight (from 93.7 kg to 73.8 kg). Cardiopulmonary and abdominal examinations were unremarkable. The BMI was reduced from obesity grade one with 30.6 kg/m² to normal weight with 24.1 kg/m² (**Table 1**).

**Table 1:** Changes in body weight, BMI, waist circumference, blood pressure and heart rate between the beginning and the end of the different stays.

Stay	01/2019		09/2022		02/2023		06/2023		09/2023		01/2024	
Parameter	Adm	End	Adm	End	Adm	End	Adm	End	Adm	End	Adm	End
Height (cm)	175	175	176	176	176	176	175	175	175	175	175	175
Body weight (kg)	93.7	86.9	92	85.2	88.4	82.2	88.7	81.3	82.1	77.1	79.5	73.8
BMI (kg/m²)	30.6	28.4	29.7	27.5	28.6	26.5	29.0	26.5	26.8	25.2	26.0	24.1
WC (cm)	117	117	114	107	109	NA	108	102	101	96	96	93
BP (mmHg)	132/81	128/85	134/83	109/75	123/77	130/85	134/90	126/85	122/76	125/86	110/60	116/73
HF (bpm)	80	66	83	77	83	66	79	72	78	65	80	71

Adm, an admission; End, on discharge; BMI, body mass index; WC, waist circumference; BP, blood pressure; HF, heart frequency; NA, not available.

In January 2019, the patient initially lost 6.8 kg, although this loss was later reversed due to recurring cravings. In 2021, his treatment was modified by adding semaglutide—titrated to 2 mg—and reducing metformin to 250 mg twice daily, which improved glycemic control and stabilized his weight; his antihypertensive medication was also discontinued at that time. By September 2022, although his general condition was good, his HbA1c remained elevated at 9.2%. In addition, in October 2022, a Fibroscan (Echosens) before fasting revealed a Controlled Attenuation Parameter (CAP) of 295 dB/m (IQR 44) and an elasticity (E) of 5.2 kPa (IQR 0.6, IQR-Med 12%), indicating S3 steatosis (67–100% liver involvement) without fibrosis (F0).

The patient then undertook repeated long-term fasting followed by periods of calorie restriction between September 2022 and January 2024 (**Figure 1**). Fasting-induced improvements in glycemic control were evident, with reductions in both fasting plasma glucose and HbA1c levels observed with each fasting cycle (**Table 2**). By May 2023, his weight had stabilized between 82 and 84 kg; however, a COVID-19 infection led to reduced activity and strong sweet cravings, causing his weight to rise to 89 kg. Despite this setback, he persisted with the protocol. In September 2023, he reported reduced hunger, prompting a temporary reduction in his semaglutide dose; however, an increase in blood sugar levels necessitated raising semaglutide back to 2 mg and increasing metformin to 500 mg.

**Table 2:** Changes in blood parameters before fasting between the different stays.

Parameter	Range	Jan-19	Sep-22	Feb-23	Jun-23	Sep-23	Jan-24
Erythrocytes (million cells/µL)	3.96-5.16	4.96	5.03	5.41	4.83	4.83	4.83
Ferritin (µg/L)	13-150	nd	149	192	135	112	95
Hematocrit (%)	35-45	42.7	42.6	47	41.3	41.9	41.2
Hemoglobin (g/dL)	11.6-15.5	13.9	14.3	15.6	13.8	14	13.8
Leukocytes (x10^3^ cells/µL)	4-10.4	6.3	6	6.7	6.6	6.7	5.5
MCH mean corposcular hemoglobin (pg)	26-33	28	28.4	28.8	28.6	29	28.6
MCHC mean corposcular hemoglobin concentration (g/dL)	32-36	32.6	33.6	33.2	33.4	33.4	33.5
MCV mean corposcular volume (fL)	80-96	86.1	84.7	86.9	85.5	86.7	85.3
Platelet (x10^3^/µL)	176-391	253	231	264	237	238	227
RDW red cell distribution width (%)	12-15	13	13	13	13	13	13
Glucose in serum (mg/dL)	60-100	184	177	97	102	105	97
HbA1c glycated hemoglobin (%)	<5.7	9.7	8.4	6.7	6.9	6.7	6.4
HOMA index (ratio)	<2.5	nd	4.7	1.6	3.6	2.4	2.6
Insulin (mlU/L)	Fasting: 2-23, long-term Fasting: <6	nd	11	6	14	9	11
INR international normalized ratio	0.85-1.15	1.02	0.96	1.13	1.13	1.07	1.06
PTT partial thromboplastin time (sec)	25.1-36.5	32	27	34	32	31	32
Quick’s test/INR prothrombin time (%)	70-130	97	107	83	83	90	92
CRP highly sensitive c-reative protein (mg/dL)	<1	1.2	1.7	1.1	1	0.4	0.31
Creatinine (mg/dL)	0.45-0.9	0.89	0.91	0.95	0.93	0.99	0.84
GFR (CKD-EPI) glomerular filtration rate (mL/min)	>90	98	95	89	91	85	97
Urea (mg/dL)	<50	21	23	29.4	21.6	24	24.4
Uric Acid (mg/dL)	<6	7.1	6.9	9.4	7.9	8.5	7.3
Cholesterol total (mg/dL)	<200	156	160	167	147	145	160
HDL-Cholesterol (mg/dL)	>48	45	49	58	52	51	58
LDL/HDL ratio	<3	2	1.9	1.7	1.6	1.4	1.7
LDL-Cholesterol (mg/dL)	<155	91	96	98	82	72	101
Non-HDL-Cholesterol (mg/dL)	<185	204	159	126	112	122	103
Alkaline Phosphatase (U/L)	35-105	74	69	66	59	57	44
Gamma-GT gamma-glutamyl transferase (U/L)	<40	44	30	29	31	19	23
GOT aspartate aminotransferase (U/L)	<35	45	24	21	18	16	12
GPT alanine aminotransferase (U/L)	<35	90	36	36	26	29	18
Calcium (mmol/L)	2.08-2.65	2.55	2.42	2.63	2.38	2.45	2.41
Magnesium (mmol/L)	0.65-1.05	0.86	0.92	0.85	0.78	0.85	0.77
Potassium (mmol/L)	3.5-5.1	5.1	4.9	5.3	4.4	4.9	4.4
Sodium (mmol/L)	132-146	141	140	140	142	143	138
TSH thyroid stimulating hormone (mE/L)	0.3-4	2.5	2.3	2.4	2.5	4	1.3
Homocysteine (µmol/L)	<10	nd	12.8	10.1	8	7.3	9.1
Vitamin B12 (ng/L)	197-771	nd	287	232	241	297	581
25-hydroxy vitamin D (µg/L)	8-56	9.9	18	13	18	27	34

During each fasting period, the patient’s antidiabetic medications at the time, including semaglutide, metformin, and glipizide, were temporarily discontinued. The antidiabetic medications were reintroduced once the daily caloric intake during food reintroduction reached approximately 1000 kcal. These adjustments were made to align pharmacological therapy with the patient’s metabolic state and nutritional intake during the fasting and food reintroduction periods.

At the end of the intervention, the patient’s HbA1c decreased from 9.7% to 6.4%. Insulin resistance estimated via HOMA index reduced from 4.7 in 09/2022 to 2.0 in 01/2024. This reflected a significant improvement in long-term blood sugar control. By January 2024, at age 57, the patient was in good general condition with only mild overweight. A follow-up Fibroscan in January 2024, showed marked improvement in liver health, with a CAP of 253 dB/m (IQR 30) and an E of 4.5 kPa (IQR 0.6, IQR-Med 13%), corresponding to S1 steatosis (5–33% liver involvement) and still no fibrosis. A subsequent Fibroscan on February 7, 2024, at the end of the last fasting period, further confirmed improvement with a CAP of 238 dB/m and an E of 2.7 kPa, maintaining S1 steatosis and no signs of fibrosis. Collectively, these results indicate significant improvements in liver steatosis over time, particularly in response to the fasting regimen. As a result of these improvements, the patient’s antidiabetic medication requirements were gradually reduced, with the eventual discontinuation of glipizide.

## 4 Metabolic changes during fasting

During the fourth visit, blood tests (**Table 3**) revealed that long-term fasting significantly reduced glucose and insulin levels. The transient increase in glucose and HOMA-IR after fasting likely reflects a temporary reduction in insulin sensitivity during the early food reintroduction phase. This phenomenon has been previously described and is thought to result from hepatic gluconeogenesis and the physiological adaptation to food reintroduction after prolonged fasting (16). Highly sensitive CRP, interleukin-6 and interleukin–8 levels also decreased markedly during fasting and food reintroduction, suggesting notable anti-inflammatory effects. Lipid levels (total cholesterol, LDL, and triglycerides) improved during fasting, in line with previously reported results (17). The patient reported improved energy, mood, and overall well-being, expressing satisfaction with the outcomes and a willingness to continue fasting.

**Table 3:** Changes in routine blood parameters during the stay in May 2023.

Parameter	Range	Baseline	End	End + 4 days	End + 14 days
Erythrocytes (million cells/µL)	3.96-5.16	4.83	5.25	4.64	4.65
Ferritin (µg/L)	13-150	135	230	188	128
Hematocrit (%)	35-45	41.3	45.7	40.1	40.2
Hemoglobin (g/dL)	11.6-15.5	13.8	14.9	13.3	13.3
Leukocytes (x10^3^ cells/µL)	4-10.4	6.6	5.1	3.9	4.7
MCH mean corposcular hemoglobin (pg)	26-33	28.6	28.4	28.7	28.6
MCHC mean corposcular hemoglobin concentration (g/dL)	32-36	33.4	32.6	33.2	33.1
MCV mean corposcular volume (fL)	80-96	85.5	87	86.4	86.5
Platelet (x10^3^/µL)	176-391	237	231	197	231
RDW red cell distribution width (%)	12-15	13	13	13	14
Glucose in serum (mg/dL)	60-100	102	81	112	119
HbA1c glycated hemoglobin (%)	<5.7	6.9	6.7	6.7	6.7
HOMA index (ratio)	<2.5	3.6	0.9	2.9	3.3
Insulin (mlU/L)	Fasting: 2-23, long-term Fasting: <6	14	4	11	11
INR international normalized ratio	0.85-1.15	1.13	1.19	1.16	1.05
PTT partial thromboplastin time (sec)	25.1-36.5	32	35	34	34
Quick’s test/INR prothrombin time (%)	70-130	83	77	81	93
CRP highly sensitive c-reative protein (mg/dL)	<1	1	0.8	0.5	0.4
Interleukin 6 (pg/mL)	<5.9	2.75	3.07	2.62	<2.50
Interleukin 8 (pg/mL)	<62	16	13	15	14
Tumor necrosis factor alpha (pg/mL)	<8.1	7	6.7	8	7.6
Creatinine (mg/dL)	0.45-0.9	0.93	1.02	0.98	1.02
GFR (CKD-EPI) glomerular filtration rate (mL/min)	>90	91	82	86	82
Urea (mg/dL)	<50	21.6	22.5	17.8	27.5
Uric Acid (mg/dL)	<6	7.9	10.2	8.9	7.5
Cholesterol total (mg/dL)	<200	147	129	108	121
HDL-Cholesterol (mg/dL)	>48	52	48	43	56
LDL/HDL ratio	<3	1.6	1.5	1.2	1.1
LDL-Cholesterol (mg/dL)	<155	82	71	53	60
Non-HDL-Cholesterol (mg/dL)	<185	95.1	81.3	65.2	65.2
Triglyceride (mg/dL)	<150	112	95	78	102
Alkaline Phosphatase (U/L)	35-105	59	71	62	59
Gamma-GT gamma-glutamyl transferase (U/L)	<40	31	26	21	23
GOT aspartate aminotransferase (U/L)	<35	18	30	25	21
GPT alanine aminotransferase (U/L)	<35	26	40	41	33
Calcium (mmol/L)	2.08-2.65	2.38	2.53	2.38	2.46
Magnesium (mmol/L)	0.65-1.05	0.78	0.86	0.83	0.83
Potassium (mmol/L)	3.5-5.1	4.4	4.7	5.1	5.2
Sodium (mmol/L)	132-146	142	141	139	137
TSH thyroid stimulating hormone (mE/L)	0.3-4	2.5	1.9	1.8	1.9
Homocysteine (µmol/L)	<10	8	9.5	6.6	9.4
25-hydroxy vitamin D (µg/L)	8-56	18	25	26	28

Blood parameters were measured at admission (adm) and for the stay in May 2023 also at the end of the fasting period (end) and during the 4th and 14th day of food reintroduction.

The patient’s gut microbiota was analyzed using shotgun metagenomics (**Table 4**). Sample collection, DNA extraction, and sequencing were conducted as described previously (see reference in preprint box), with composition determined using MetaPhlAn 4.1. A total of 149 bacterial species were identified. The microbiota rapidly shifted from being dominated by bacteria that utilize dietary substrates to those that exploit host-derived substances, as observed in previous studies (18, 19). Remarkably, within 14 days the microbiota returned to its baseline composition, demonstrating significant resilience to the dietary intervention. After three months, no discernible differences were observed, and the microbiota maintained its individual characteristics.

**Table 4:** Impact of fasting on faecal microbiota profiles.

Bacteria species	Baseline	End of fasting	End + 14 days	End + 3 months
Bacteroides uniformis	22.9%	1.9%	23.5%	17.5%
Phocaeicola plebeius	12.8%	7.3%	6.9%	8.7%
Brotolimicola acetigignens	10.8%	0.0%	3.4%	5.5%
Faecalibacterium prausnitzii	6.9%	0.4%	6.6%	8.3%
Bacteroides ovatus	6.7%	2.3%	7.2%	6.9%
Phocaeicola vulgatus	5.4%	9.4%	9.2%	5.1%
Ruminococcus callidus	2.9%	0.0%	0.0%	3.2%
Bacteroides nordii	2.6%	4.4%	0.6%	0.4%
Blautia wexlerae	1.6%	0.1%	1.9%	2.7%
Dialister succinatiphilus	1.5%	0.0%	2.0%	1.2%
Bacteroides thetaiotaomicron	1.4%	3.3%	1.1%	1.4%
Fusicatenibacter saccharivorans	1.2%	0.0%	1.3%	2.5%
Bacteroides caccae	1.2%	17.8%	1.2%	1.0%
Blautia faecis	1.1%	0.2%	0.3%	1.3%
Anaerostipes hadrus	0.9%	0.2%	1.5%	2.6%
Bifidobacterium longum	0.8%	0.0%	0.5%	2.0%
Sutterella wadsworthensis	0.7%	1.4%	1.6%	0.6%
Anaerobutyricum hallii	0.7%	0.1%	1.4%	1.0%
Odoribacter splanchnicus	0.7%	2.2%	0.8%	0.6%
Faecalibacterium SGB15346	0.6%	0.2%	1.3%	0.6%
Bacteroides xylanisolvens	0.6%	0.1%	1.8%	0.7%
Candidatus Cibionibacter quicibialis	0.6%	0.0%	0.4%	1.4%
Agathobaculum butyriciproducens	0.5%	0.0%	1.1%	0.7%
Dysosmobacter welbionis	0.5%	4.8%	0.4%	0.3%
Alistipes shahii	0.4%	1.6%	1.4%	0.3%
Parabacteroides merdae	0.4%	2.1%	0.6%	0.2%
Pseudoflavonifractor capillosus	0.3%	2.6%	0.7%	0.2%
Intestinimonas butyriciproducens	0.3%	7.4%	0.4%	0.1%
Flavonifractor plautii	0.2%	4.1%	0.2%	0.1%
Bifidobacterium catenulatum	0.2%	0.0%	0.3%	4.9%
Bilophila wadsworthia	0.2%	1.1%	0.4%	0.2%
Ruminococcus gnavus	0.1%	2.0%	0.0%	0.1%
Blautia sp MCC283	0.1%	0.0%	1.9%	1.8%
Enterocloster bolteae	0.0%	1.7%	0.1%	0.0%
Streptococcus thermophilus	0.0%	1.5%	0.0%	0.2%
Ruthenibacterium lactatiformans	0.0%	1.0%	0.1%	0.0%
Roseburia intestinalis	0.0%	0.0%	0.0%	1.5%

Shotgun metagenomics analyses were conducted during stay 4 (05/2023) and 3 months later at the baseline of stay 5 (09/2023). Only bacteria with relative abundance over 1% at any timepoint are shown.

## 5 Discussion

This case report highlights the potential benefits of repeated long-term fasting for improving glycemic control and reducing medication requirements in patients with T2DM. The observed effects on weight reduction, insulin sensitivity, and lipid metabolism are consistent with previous studies investigating the metabolic effects of fasting in diabetic individuals (2, 8).

Fasting-induced weight loss is believed to improve insulin sensitivity by reducing ectopic fat accumulation and adipose tissue inflammation, thereby enhancing glucose uptake and utilization by peripheral tissues (2). Moreover, fasting promotes autophagy and mitochondrial biogenesis, processes that contribute to cellular repair and metabolic homeostasis (20).

The progressive amelioration of glycemic control over time observed in our patient is likely attributable to a combination of factors, including decreased hepatic glucose output, increased insulin sensitivity, and enhanced pancreatic beta-cell function, and, to a large extent, overall weight loss. Fasting-induced ketosis may also play a role in glucose regulation by providing an alternative energy substrate for tissues (6), thereby reducing reliance on glucose.

The anti-inflammatory effects of fasting, evidenced by reductions in hs-CRP levels, are of particular relevance in the context of T2DM, as chronic low-grade inflammation is implicated in the pathogenesis of insulin resistance and metabolic dysfunction (21).

Dietary choices can affect quality of life, including fatigue and emotional well-being (22, 23), support male reproductive health (24), and modulate sleep via effects on circadian and metabolic processes (25).

While the findings of this case report are promising, several limitations should be noted. Firstly, as a single case report, generalizability to broader populations may be limited. Additionally, the long-term safety and efficacy of repeated long-term fasting in T2DM require further investigation through randomized controlled trials and longitudinal studies. Close monitoring of patients undergoing fasting interventions is essential to mitigate potential risks, including hypoglycemia, electrolyte imbalances, and nutrient deficiencies.

## 6 Perspective of the patient

Think of being on a bike and going down on an incline. At first you can slow down or even brake but slowly you’ll keep going down. Gravity makes it. This is what it was like for me seeing multiple generations on my mother’s side suffering from diabetes. Each and every one of them gained weight, all had diabetes and follow-on health issues. All of them went on diabetes medication insulin with dosage increasing over time. Their weights also went up; gradually at first but then faster and faster. Health, wellness and overall liveliness suffered because of diabetes—an incurable disease which could be marginally controlled but never cured.

I was headed on that same path as I was diagnosed with diabetes earlier. I used existing medical treatments but all of the medical professionals kept telling me that I needed to lose weight—prescribing some version of “eat less move more” along with a smattering of non-insulin medications. I tried those suggestions but my condition got worse and I was getting closer to having to be on insulin.

The problem? Near-constant voice inside my head about being hungry and hungry for the wrong kinds of foods. Non-diabetics would say I had no self control. Which is interesting as I don’t lie, cheat or steal but apparently only when it comes to food, my self restraint vanishes?

Out of desperation, I visited a fasting clinic in 2019. At the end of that visit, I lost nearly 7 kilograms of weight as well as abdominal fat. My excitement over the weight loss was short-lived. Within barely two months, I gained it all back. Two months!

While this reversal of weight loss was a negative, in hindsight, it was the basis of my preparation for the next five visits.

Determined to have better outcome during my follow-up visits, I started reading up on diabetes, weight loss, microbiome and hunger. Based on these plus my experience, I decided on the following basics: Weight loss of 15 to 20% was needed to reverse my diabetes. Dr. Roy Taylor of University of New Castle’s excellent research proved it. Longer food reintroduction periods after fasts so I don't slide back into constant hunger. Also, to ask for help from my wife during these transitions.

At the time, no exact protocol for what I was trying to do. So, there was the minor risk of the unknown of multiple fasts in short intervals. A chance encounter with Dr. Françoise Wilhelmi de Toledo who spoke with Dr. Mesnage got this research started.

Fasting works. I know because I’ve tried to fast at home and that is tough! At the fasting clinic, I felt the whole place was geared toward gently guiding me through to weight loss but not have me starve for food.

But the part that’s just as important for me was the food reintroduction period after the long fasts. The billions of my gut bacteria eat what I eat. If the reason my gut bacteria ask for wrong types of food could be that the good bacteria are overcome by the bad. If so, then a diverse group of the foods that are fuel for the good bacteria specially in the food reintroduction stage will be needed. A longer food reintroduction time will help them grow.

As I went through a few fasting and long food reintroduction sessions, my relationship with food changed. Previously, during fasting periods, I’d day dream of unhealthy options for food. But soon during my fasting period, my day dreams started to change—I would dream of roasted cauliflowers with olive oil, salt and black pepper.

Now the difference was not dramatic but grew over time, I started noticing that I’m less and less hungry. Healthier food options were satisfying and no, I did not have to use some Jedi mind trick to feel this way. This does not mean I don’t have occasional hankering for some bad food … I do. But as my visits to the fasting clinic progressed, the occurrences became few and far between.

## 7 Conclusion

In conclusion, repeated long-term fasting with food reintroduction may represent a promising strategy for inducing weight loss, improving glycemic control and reducing diabetes medication requirements, as observed in our patient affected by type 2 diabetes mellitus (T2DM) and obesity. The observed effects on metabolic parameters, including blood glucose levels, lipid profiles, and markers of inflammation, underscore the potential metabolic benefits of fasting in diabetic individuals. However, fasting interventions should always be conducted under medical supervision and tailored to individual needs to ensure safety and tolerability. Further research is warranted to elucidate the long-term effects, safety profile, and optimal fasting regimens for managing T2DM and related metabolic disorders in randomized controlled trials.

## Data Availability

The original contributions presented in the study are included in the article/supplementary material, further inquiries can be directed to the corresponding author/s.
